# Drug resistance to sulphadoxine-pyrimethamine in *Plasmodium falciparum *malaria in Mlimba, Tanzania

**DOI:** 10.1186/1475-2875-5-94

**Published:** 2006-10-31

**Authors:** Erasto V Mbugi, Benezeth M Mutayoba, Allen L Malisa, Sakurani T Balthazary, Thomas B Nyambo, Hassan Mshinda

**Affiliations:** 1 Department of Veterinary Physiology, Biochemistry, Pharmacology and Toxicology, Faculty of Veterinary Medicine, Sokoine University of Agriculture (SUA), P.O. Box 3017, Morogoro, Tanzania; 2 Department of Biological Sciences, Faculty of Science, Sokoine University of Agriculture, P.O. Box 3038, Morogoro, Tanzania; 3 Department of Biochemistry, School of Medicine, Muhimbili University College of Health Sciences (MUCHS), P.O. Box 65001, Dar es Salaam, Tanzania; 4 Ifakara Health Research and Development Centre (IHRDC), Off Mlabani Road, P.O. Box 53, Ifakara, Kilombero District, Morogoro, Tanzania; 5 Cell Biology and immunology group, Department of Animal Sciences, Wageningen University, P.O. Box 338, 6700 AH Wageningen, The Netherlands

## Abstract

**Background:**

Sulphadoxine-pyrimethamine (SP) has been and is currently used for treatment of uncomplicated *Plasmodium falciparum *malaria in many African countries. Nevertheless, the response of parasites to SP treatment has shown significant variation between individuals.

**Methods:**

The genes for dihydrofolate reductase (*dhfr*) and dihydropteroate synthase (*dhps*) were used as markers, to investigate parasite resistance to SP in 141 children aged less than 5 years. Parasite DNA was extracted by Chelex method from blood samples collected and preserved on filter papers. Subsequently, polymerase chain reaction (PCR) and restriction fragment length polymorphism (PCR-RFLP) were applied to detect the SP resistance-associated point mutations on *dhfr *and *dhps*. Commonly reported point mutations at codons 51, 59, 108 and 164 in the *dhfr *and codons 437, 540 and 581 in the *dhps *domains were examined.

**Results:**

Children infected with parasites harbouring a range of single to quintuple *dhfr*/*dhps *mutations were erratically cured with SP. However, the quintuple *dhfr*/*dhps *mutant genotypes were mostly associated with treatment failures. High proportion of SP resistance-associated point mutations was detected in this study but the adequate clinical response (89.4%) observed clinically at day 14 of follow up reflects the role of semi-immunity protection and parasite clearance in the population.

**Conclusion:**

In monitoring drug resistance to SP, concurrent studies on possible confounding factors pertaining to development of resistance in falciparum malaria should be considered. The SP resistance potential detected in this study, cautions on its useful therapeutic life as an interim first-line drug against malaria in Tanzania and other malaria-endemic countries.

## Background

Human malaria is caused by an Apicomlexan parasite of the genus *Plasmodium*. Four species are known to cause human malaria namely, *Plasmodium falciparum*, *Plasmodium vivax*, *Plasmodium ovale *and *Plasmodium malariae*. Nevertheless, *P. falciparum *has been found to be the most lethal of all human malaria parasites. This parasite causes epidemics in malaria-endemic countries, resulting in large numbers of deaths. Widespread chloroquine resistance has forced many countries to use alternative drugs as antimalarials against falciparum malaria, such as the combination of sulphadoxine and pyrimethamine (SP). However, the parasite has been observed to develop resistance to this drug combination associated with point mutations in the genes for the enzymes involved in the obligatory parasite-folate biosynthesis pathway. Such mutations lead to the lowering of the drug binding affinity to the parasite enzymes [1-5]. Resistance to pyrimethamine is attributed to mutations in the gene for the parasite enzyme dihydrofolate reductase (*dhfr*), whereas sulphadoxine resistance is associated with mutations in the gene for the parasite enzyme dihydropteroate synthetase (*dhps*). The increased level of resistance has been found to be associated with increased numbers of mutations in the genes for these two enzymes. Studies [6] have shown that multiple mutations in the genes for both enzymes result in exceedingly high SP treatment failure. Detection of these mutations in field isolates has been proposed as an alternative strategy for rapid screening of antifolate drug resistance [7-12].

In Tanzania, due to high resistance that developed against the previously effective, safe and relatively cheap antimarial drug, chloroquine, SP was introduced as the first-line drug against malaria by August, 2001. Unfortunately, the change of policy to SP by the government has been challenged by the previously reported low [13] but fast spreading levels of resistant parasite strains against the drug [14]. SP resistance has been reported in variable magnitudes across the country [15-17]. Surveillance for these antifolate-resistant parasites in the field is still required to dissuade its spread over wide areas and possibly suggest effective implementation of new drug policy in Tanzania. The present study was thus carried out during the period of January 2002 to August 2004 to evaluate the frequency of point mutations in *dhfr *and *dhps *among *P. falciparum *isolates from children of Mlimba division of Kilomero district of Tanzania. This could give a picture of the level of drug pressure in the field from the time when SP was introduced as an interim first-line drug for malaria treatment in the country. Since there are currently, various drug combinations on trial for treatment of uncomplicated falciparum malaria [18,19], the anticipation was to obtain findings which would give information on the current frequencies of SP resistant *P. falciparum *strains and probably give advice to policy makers for opting to other new effective, cheap and safe antimalarial drug combinations.

## Materials and methods

### Study area

The study was conducted at Ifakara Health Research and Development Center (IHRDC) situated in Ifakara Town of Kilombero District, Morogoro, Tanzania. Samples were collected during the period of January to August 2002 from Mlimba, an area about 150 km from IHRDC along Kilombero River where malaria is endemic with perennial transmission. The area is among nine sentinel sites for National Malaria Control Programme since 1997 and its human population dynamics is being closely monitored on a monthly basis by the Ifakara Centre Demographic Surveillance System (IC-DSS) since 1996. Recruitment of patients and sample collection was done by the research team at Mlimba Health Centre.

### Study subjects

The ethical clearance was obtained from both National Institute for Medical Research (NIMR) and IHRDC Institutional Ethics Committee authorities. Parents or guardians of participating children accepted and gave informed consent for participation in the study. About 172 patients of both sexes with acute uncomplicated falciparum malaria and aged 6 – 69 months (< 5 years) were initially recruited in this study. However 31 (18%) of the recruited patients either were excluded from the study due to failure to comply with criteria for participating in the study or were lost during follow up.

### Sample collection

Blood samples for parasite genotyping were collected on filter paper (3 MM Whatman), labelled and identified, and kept in a dry clean container with desiccant for a minimum of three hours to dry. Dry filter paper blood samples were stored at room temperature until when needed for further analyses. The follow-up samples were obtained at days 3, 7 and day 14 after SP treatment. Additional follow-ups were done at any other day if the child was unwell. During all these visits, finger-prick blood was obtained for microscopy and later molecular analysis.

### Extraction of parasite DNA

*P. falciparum *genomic DNA was extracted from blood collected on 3 MM Whatmann filter paper by Chelex method as previously described [20]. The extracted DNA from each sample was used immediately for PCR and any remaining portion was stored at -20°C in appropriately labelled storage tubes.

### Genotyping of parasite genomic DNA

Sample analysis was based on the standardised polymerase chain reaction and restriction fragment length polymorphism. For amplification of the *dhfr *and *dhps *coding regions, a nested PCR protocol was adopted followed by RFLP. The regions of the *dhfr *and *dhps *genes surrounding the polymorphisms of interest in *dhfr *51, 59, 108 and 164 and *dhps *437, 540 and 581 codons were analysed as described in detail elsewhere [7,21,22].

### Amplification of parasite DNA by PCR

In this multiplex parasite DNA amplification of the parasite genomic DNA, two primer pairs M1/M5 and R2 + R/ were used as forward and reverse primers in primary (nest I) PCR reaction for the *dhfr *and *dhps *domains respectively. In secondary (nest II) PCR reaction M3 + F/ and F + M4 were used as forward and reverse primers to amplify the four regions on *dhfr *where the point mutation is anticipated to occur [20]. On the other hand, K +K/ and L + L/ primers were used to amplify regions on the *dhps *gene where resistance-associated mutations are said to occur [7]. The details of primer sequences, annealing temperatures and controls are shown in Table 1. In both nest I and nest II PCR, reaction volumes ranged from 20 μl to 30 μl. The final concentration of each reagent was 1× PCR reaction buffer (10× PCR buffer – MgCl_2_, Invitrogen), 1.5 mM MgCl_2_, 125 μM dNTP (Promega, Madison, WI, USA), 250 nM primers (QIAGEN, Operon) and 0.02 U/μl Taq Polymerase (Invitrogen). The master mix was prepared in a 1.5 ml reaction tube with Molecular biology PCR water (Sigma) as a diluent and aliquots made in PCR tubes (0.2 ml size). To each PCR tube, 5 μl of DNA was added in primary reaction and 2 μl was re-amplified in the nested PCR reaction. The known purified genomic DNA from HB3, 3D7, W2, K1, T9/96, FCR3 and V1/S laboratory parasite clones were used as positive controls and NT (No template) was included as negative control. PCR was performed in a Programmable Thermo Controller, (PTC-100 (TM) MJ Research, Inc., Watertown, MA, USA). Samples with no detectable PCR products were re-examined at least twice starting from the DNA preparation before were declared negative.

**Table 1:** Polymorphic genes investigated, mutation sites, primers and primer sequences, PCR reaction conditions, restriction enzymes and control DNA used in this study

**Gene**	**Mutation**	**Primers**	**Primer Sequences**	**PCR conditions**	**Enzyme digest**	**Control DNA**
DHFR Primary		M1 M5	5' TTTATGATGGAACAAGTCTGC3' 5' AGTATATACATCGCTAACAGA3'	94°C-3 min, 94°C-1 min, 50°C-2 min, 72°C-2 min, ×40, 72°C-10 min, 4°C-hold		
DHFR Nested	N51I	M3 F/	5'TTTATGATGGAACAAGTCTGCGACGTT3' 5'AAATTCTTGATAAACAACGGAACCTttTA3'	94°C-2 min, 94°C-1 min, 45°C-1 min, 72°C-2 min, ×35, 72°C-10 min, 4°C-hold	*TSP509*1	(+) K1
	C59R	F M4	5'GAAATGTAATTCCCTAGATATGGAATATT3' 5'TTAATTTCCCAAGTAAAACTATTAGAGCTTC3'	94°C-2 min, 94°C-1 min, 45°C-1 min, 72°C-2 min, ×35, 72°C-10 min, 4°C-hold	*Xmn*I	(+) T9/96
	S108N	F M4	5'GAAATGTAATTCCCTAGATATGGAATATT3' 5'TTAATTTCCCAAGTAAAACTATTAGAGCTTC3'	94°C-2 min, 94°C-1 min, 45°C-1 min, 72°C-2 min, ×35, 72°C-10 min, 4°C-hold	*Alu*1	(+) HB3 (+) T9/96 (+) FCR3
	I164L	M3 F/	5'TTTATGATGGAACAAGTCTGCGACGTT3' 5'AAATTCTTGATAAACAACGGAACCTTTTA3'	94°C-2 min, 94°C-1 min, 45°C-1 min, 72°C-2 min, ×35, 72°C-10 min, 4°C-hold	*Dra*I	(+) V1/S (+) HB3 (+) DD2
DHPS Primary		R2 R/	5' AACCTAAACGTGCTGTTCAA3' 5' AATTGTGTGATTTGTCCACAA3'	94°C-3 min, 94°C-1 min, 50°C-2 min, 72°C-2 min, ×40, 72°C-10 min, 4°C-hold		
DHPS Nested	A437G	K K/	5'TGCTAGTGTTATAGATATAGGATGAGcATC3' 5'CTATAACGAGGTATTGCATTTAATGCAAGAA3'	94°C-2 min, 94°C-1 min, 45°C-1 min, 72°C-2 min, ×35, 72°C-10 min, 4°C-hold	*Ava*II	(+) K1 (+) FCR3
	K540D	K K/	5'TGCTAGTGTTATAGATATAGGATGAGCATC3' 5'CTATAACGAGGTATTGCATTTAATGCAAGAA3'	94°C-2 min, 94°C-1 min, 45°C-1 min, 72°C-2 min, ×35, 72°C-10 min, 4°C-hold	*Fok*I	(+) TN-1 (+) VIS (+) W2
	A581G	L L/	5'ATAGGATACTATTTGATATTGGACCAGGATTCG3' 5'TATTACAACATTTTGATCATTCGCGCAACCGG3'	94°C-2 min, 94°C-1 min, 45°C-1 min, 72°C-2 min, ×35, 72°C-10 min, 4°C-hold	*Bst*UI	(+) K1 (+) DD2

**Source: **Duraisingh et al. [21]; Foley et al. [48]

### Restriction enzyme digestion

Site-specific restriction enzymes were used to digest the PCR amplicons. Seven different restriction enzymes were used in this study (Table 1), namely *TSP509*I, *Xmn*I,*Alu*I, *Dra*I (*dhfr *domain) and *Ava*II, *Fok*I, *Bst*UI (*dhps *domain) enzymes, respectively. Essentially, 8 μl of PCR products were incubated with restriction enzymes (New England Biolabs, Beverly, MA, USA) according to manufacturer's protocol in 25 μl final reaction volume. The *dhfr *and *dhps *variants were identified as previously described [7,21,23].

### Gel electrophoresis

Nested PCR amplicons were electrophoresed on 2% agarose gels before subsequent restriction fragment length polymorphism analysis. Electrophoresis of restriction digests was done on 10% polyacrylamide gel (PAA) as described by Sambrook et al. [24] at a constant voltage of 11.25 v/cm gel for 2.30 hours, stained with ethidium bromide, visualized under UV light, photographed and electronically stored.

### Statistical analysis

Data were analysed using the EPI Info Version 6.04 epidemiological software (Centres for Disease Control and Prevention, Atlanta, GA, USA). This made it possible to estimate the frequency of point mutations on *dhfr *and *dhps *responsible for parasite resistance against SP thus determining the prevalence of these mutations. The prevalence of each point mutation was calculated as the percentage of baseline (D0) samples containing point mutation at the particular codon on *dhfr *and *dhps*, respectively. Fragment sizes were compared with known restriction fragments (band sizes) obtained in previous studies with reference to 1 Kb DNA marker [21,25].

## Results

### Treatment outcomes

From clinical evaluation (clinical data provided by IHRDC), a total of 172 children with acute uncomplicated malaria were recruited into the study and treated with SP. Out of these recruited patients, 141 (82%) successfully completed the study. Data from 31 (18%) patients who could not complete the 14 days follow-up were excluded from analysis. Of patients who completed the study successfully, treatment failures were depicted in 15 (10.6%) patients comprising of 6.7% early and 3.9% late treatment failures, respectively. Adequate clinical response occurred in 126 (89.4%) of patients. Consequently, molecular analysis was performed on 141 samples from patients who completed the study.

### PCR amplification of *dhfr *and *dhps*

**Figure 1:**
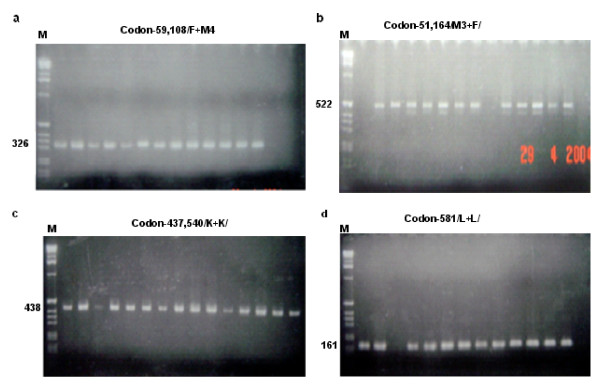
Agarose gels of the PCR products ((a) F+M4; (b) M3+F/; (c) K+K/; (d) L+L/) of the tests for the polymorphisms of *dhf *and *dhps*. Fragment sizes are in bp.

Different primers were used to amplify regions in *dhfr *and *dhps *containing various point mutations associated with resistance to SP, thus different DNA fragments with different band sizes were obtained (Figure 1). The fragment sizes were estimated as previously described elsewhere [21]. F + M4 amplification produced DNA amplicons of about 326 bp (Figure 1a) while DNA amplicons of approximately 522 bp band size were obtained following DNA amplification by M3 + F/ primers (Figure 1b). PCR amplification using K + K/ (Figure 1c) and L + L/ primers (Figure 1d) produced fragments of 438 and 161 bp, respectively on 2% agarose gel. Of 141 samples analysed, 120 (85.1%), 136 (96.5%), 132 (93.6%) and 133 (94.3%) were successfully amplified using M3+ F/, F + M4, K + K/ and L +L/ primers, respectively.

### RFLP analysis

**Figure 2:**
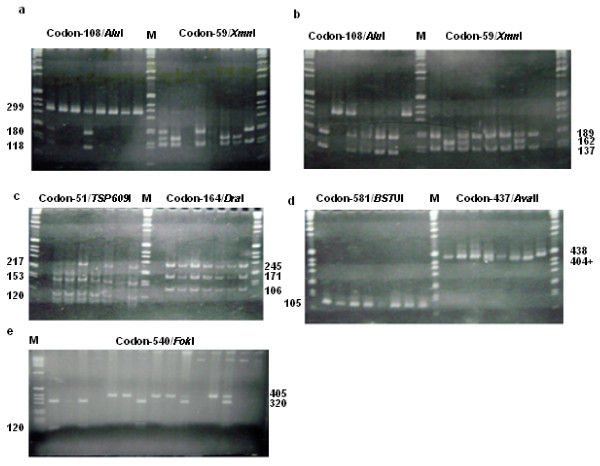
Polyacrylamide gels of the restriction digest of the PCR products ((a)*Alu*I and *xmn*I; (b) *Alu*I and *xmn*I; (c)*TSP609*I and *Dra*I; (d) *BSTU*I and *Ava*II; (e) *Fok*I) of the tests for the polymorphisms of the *dhfr *and *dhps *genes. Fragment sizes are in bp.

Figure 2 depicts representative PAA gels of the restriction digests used to detect the fragment patterns corresponding to the different variants at each codon on the *dhfr *and *dhps *domains. PCR amplification of *dhfr *with the primers F + M4 yields a PCR product sized 326 bp of the codon 108. The restriction enzyme *Alu*I was used specifically to cut fragments discriminating the two alternative forms, wild-type (180 and 118 bp) and mutant (299 plus 27 bp) at that codon. The wild type indicates presence of serine (Ser) in the amino acid sequence of the enzyme system while the mutant form of the gene indicates the substitution by asparagine (Asn). Restriction digestion of the same PCR fragment with *Xmn*I was used to distinguish wild type (189 and 137 bp) and mutant (162, 137 + 27 bp fragments) variants on codon 59 of the gene. Mutation at this codon reflects substitution of amino acid cystine (Cys, wild type) by arginine (Arg, mutant).

The M3 + F/ amplified PCR fragments (Figure 1b) were digested with *TSP509*I and *Dra*I to distinguish variants at codon 51 and 164, respectively. Digestion by *TSP509*I produced fragments of sizes 153 and 120 bp indicative of presence of amino acid asparagine (wild type) and 217 and 120 bp indicating presence of amino acid isoleucine (Ile, mutant). On the other hand, digestion with *Dra*I was expected to produce fragments of sizes 245, 171 and 106 bp (wild type) and 145, 143, 106 and 28 bp (mutant) distinguishing polymorphisms at codon 164. In this context wild type denotes occurrence of isoleucine (Ile) and the mutant indicates occurrence of leucine (Le). The four restriction enzymes were used mutually to detect mutations on the *dhfr *gene ascribed to resistance to antifolate, SP by the malaria parasite.

Tests for polymorphisms on *dhps *are shown in Figures 2d and 2e. PCR products of approximately 438 bp (Figure 1c) were obtained from amplification with the K and K/ primers. The variants at codon 437 were discriminated by restriction digestion using the restriction enzyme *Ava*II (Figure 2d). In this case uncut fragment (438 bp) indicated wild type while mutations at this site was shown by a cut fragment sized 404+ bp. Mutation at this codon is indicative of substitution of glycine (Gly) for alanine (Ala). The K + K/ amplified PCR products have been used to detect polymorphisms occurring at codon 540. A restriction enzyme, *Fok*I produced fragments sized 405 bp (wild type) and 320 and 85 bp (mutant) discriminating variants at that codon. The 405 bp fragment indicates presence of amino acid Lysine (Lys) while the 320 and 85 bp fragments reflect substitution of glutamate (Glu) for lysine at this codon.

Following amplification with L + L/ primers, PCR products of size 161 bp were produced (Figure 1d). These PCR fragments have been used to describe polymorphism on the *dhps *gene occurring at codon 581 using a restriction enzyme *Bst*UI. This restriction enzyme produced restriction digests of sizes 105, 33 and 23 bp (wild type) and 138 and 23 bp (mutant) from the PCR products of size 161 bp (Figure 2d). The former reflects presence of amino acid, alanine at this codon and the latter indicates substitution of glycine for alanine.

Mutation analysis was variably successful at each codon in both *dhfr *and *dhps *genes and an average of 120 (85.1%) of 141 patients produced fruitful outcome. Four codons (51, 59, 108 and 164) on *dhfr *and three codons (437, 540 and 581) on *dhps *were evaluated and the results are presented in Figures 2. Mutations in the *dhfr *were highest for Asn-108 (66.9%) and progressively declined for *dhfr *Ile-51 (62.7%) and *dhfr *Arg-59 (48.8%). Mutations in *dhps *varied from *dhps *Gly-437 (43.7%), *dhps *Glu-540 (39.2%) and was lowest for *dhps *Gly-581 (0.8%) alleles. In all pre-treatment and post-treatment samples, no mutant *dhfr *Leu-164 was depicted. The triple *dhfr*, double *dhps *and the quintuple mutants (carrying the *dhfr *triple mutant and the *dhps *double mutant) were also evaluated and were considerable (Table 3). Mixed pattern was not uncommon in many samples examined (Table 4).

**Table 2:** Proportions of point mutations on *dhfr *and *dhps *related to parasite resistance to antifolates

Resistance to	Gene locus	Mutations (%)	Wild type (%)	Mixed (%)	Total
Pyrimethamine	DHFR 51	74 (62.7%)	35 (29.7%)	9 (7.6%)	118
	DHFR 59	59 (48.8%)	36 (29.8%)	26 (21.5%)	121
	DHFR 108	81 (66.9%)	17 (14.1%)	23 (19.0%)	121
	DHFR 164	0 (0.0%)	104 (100.0%)	0 (0.0%)	104
Sulphadoxine	DHPS 437	56 (43.7%)	71 (55.5%)	1 (0.8%)	128
	DHPS 540	47 (39.2%)	65 (54.5%)	8 (6.3%)	120
	DHPS 581	1 (0.8%)	126 (98.4%)	1 (0.8%)	128
		318	454	68	840

**Table 3:** Various *dhfr*/*dhps *combinations obtained from restriction analysis to determine point mutations in genes responsible for SP resistance.

Category	Number	Percentage (%)	Percent Cum.
tDHFR	44	36.40	36.40
dDHPS	18	14.90	51.30
tDHFR/dDHPS	24	19.80	71.10
dDHFR/dDHPS	10	8.30	79.40
tDHFR/sDHPS	4	3.30	82.70
dDHFR/sDHPS	7	5.80	88.50
tDHFR/nDHPS	14	11.50	100.00
Total	121	100.0	

tDHFR = Triple mutants on *dhfr *gene; dDHFR = Double mutants on *dhfr *gene; dDHPS = Double mutants on the *dhps *gene; sDHPS = Single mutant on the *dhps *gene; nDHPS = No mutation on the *dhps *gene

**Table 4:** Proportions of mixed infections detected in this study

Category	Variants detected	Total No. of variants	Percentage (%)
smDHFR	27 × 1	27	39.7
smDHPS	8 × 1	8	11.8
dmDHFR	8 × 2	16	23.5
dmDHPS	1 × 2	2	2.9
tmDHFR	5 × 3	15	22.1
tmDHPS	0 × 3	0	0.00
Total		68	100

smDHFR = Mixed variants detected at a single codon on the *dhfr *domain; mDHPS = Mixed variants detected at a single codon on the *dhps *domain; dmDHFR = Mixed variants detected at two codons on the *dhfr *domain; dmDHPS = Mixed variants detected at two codons on the *dhps *domain; tmDHFR = Mixed variants detected at three codons on the *dhfr *domain; tmDHPS = Mixed variants detected at three codons on the *dhps *domain

## Discussion

The 141 patients represent children aged less than five years, the age most vulnerable to falciparum malaria [26]. Parasite recurrence in SP treated individuals has been linked to many factors. Such factors include overwhelmed immunity, multiple concurrent infections and drug resistance [27]. Several previous studies have investigated the association between mutations in *dhfr *and *dhps *and the parasitological and/or clinical response to SP medication at individual level [6,28-30]. Most of these studies produced tangible results regarding the use of *dhfr *and *dhps *genotypes as resistance marker genes for SP [31]. In this study, it was observed (clinical data) that SP treatment cleared infection in 89.4% of the patients who completed the study. This treatment success is partly concordant but higher than what has previously been reported (82%) in a similar study by Aubouy et al. [25] in Bakoumba village in Haut-Ogooué province of Southeast Gabon. According to that study, *dhfr *mutations that lead to high-level *in vitro *resistance to pyrimethamine plus one or two *dhps *mutations were reported to be not sufficient to induce *in vivo *failure of SP treatment in young children. Nevertheless, the semi-immune population of over 60% previously reported in Tanzania [32] is probable reminiscent for the observed high parasite clearance despite detection of high resistance-associated point mutations in this study.

Slightly higher values of mutations on *dhfr *(66.9% and 62.7%) were detected at codons 108 and 51, respectively as compared to those previously reported by Mshinda [33] of 50%. This is attributable to rapid, stepwise selection of mutations following use of antimalarial, SP or similar drugs in the area. This is simply because our study was carried 2 years after a similar study by Mshinda in 2000. Pharmacological case management of the disease occurring at informal level [26,34] with poor compliance with dosing schedules could be another basis for the high frequency of mutations detected in this study. In addition, sub-dosage levels of drug administration e.g. two patients sharing a single dose prescribed for a single patient (personal observation) may be a probable cause for the detected high proportion of mutations. Extensive use of different types of antifolates with mechanism of action similar to SP like trimethoprim and sulphamethoxazole (TS) combination (e.g. septrin) in treating other infections over time, probably accounts for the increased selection of mutations ascribed to SP resistance [17].

The mutation of about 66.9% observed in *dhfr *on codon 108 was higher than that reported by Jelinek et al. [35] in West Africa (54.0%) but slightly lower than that observed in Central Africa (72.4%), South Africa (68.9%) and East Africa (72.9%). This could be correlated to the high proportion of mixed genotype infections (mutant and wild type, 19.0%) detected at this codon in the present study. Pearce et al. [17] reported the increase of mixed genotypes in codons whose mutations are associated with SP resistance in an area of high endemicity. This proportion of mixed genotypes could give rise to *dhfr *mutations of about 85.9% if at all these genotypes were additive. The *dhfr *point mutations at codons 51 and 59 were also higher than that previously reported by Jelinek et al. [35] in Africa and all together explains the significance of these mutations in causing higher SP resistance. As previously reported by Hastings et al. [36], mutations at codon 164 in *dhfr*, which is thought to confer highest SP resistance when it occurs, was not depicted in this study. Generally, mutations in the three codons in the *dhfr *domain were higher (Table 2) than those on the *dhps *domain, suggesting that mutations on *dhfr *precede those on *dhps *in conferring parasite resistance to SP [37].

The point mutations in *dhps *depicted in this study are far higher than what was observed in previous studies in East and South Africa [35] and Eastern Iran [38]. This might be explained by the widespread use of septrin in Tanzania, which is said to indirectly select mutations for SP resistance [17]. Takahashi et al. [39] further reported that the use of antifolates such as co-trimoxazole for prophylaxis or medication against other infections than malaria indirectly and predominantly select double mutations at *dhps *for resistance. Nevertheless, the *dhps *mutations observed in this study were relatively lower than that observed in Central and West Africa by Jelinek et al. [35], possibly due to geographical differences [16] and differences in patterns of drug use between different areas [17]. However, Beswas [16] reported the *dhps *mutations in Tanzania ranging between 30 – 34%, which is less than values observed in our study (39.2 – 43.7%) signifying that drug pressure increasingly selects these mutant alleles with time depending on frequent use of antifolates.

Overall proportion of point mutations portrayed in this study reflects the existence of high resistance in the study area. Only 8.5% of infections carried pure wild type genotype and 4.6% of the samples showed single mutant alleles. Quintuple mutations were highest (18.5%) followed by triple (16.2%) and double mutations (13.8%). Quadruple mutations occurred in 6.9% of samples. About 41 (31.5%) of infections had at least one mixed (mutant, wild type) genotypes attributable to endemicity of the disease [40], which may result into high transmission consequently augmenting high proportion of mixed clones per infection in the population.

The co-occurrence of point mutations in both *dhfr *and *dhps *loci was also examined in this study (Table 3) with about 44 (36.4%) of samples containing triple mutant on *dhfr*, which was relatively similar to that reported by Mshinda [33] in the same place (41%) but was almost twice to values reported by Mugittu et al. [15] of 18.6%. However, the proportion could escalate if the mixed triple *dhfr *variants (22.1%) and mixed double *dhfr *variants 16 (23.53%) detected in the present study (Table 4) were counted inclusively. Nevertheless, the proportion of these mutations was less than that reported by Pearce et al. [17] in Hai district Northern Tanzania (>70%) where resistance to SP is already unprecedentedly high due to widespread use of SP and spread of resistance from nearby area, Muheza into the area. Mugittu et al. [15] reported a prevalence of 80.3% in Mkuzi, an area in Muheza District where pyrimethamine was used for prophylactic and/or therapeutic trials at different periods from 1950s to 1994 [41-43]. The difference in levels of SP resistance between Tanzanian communities is also attributable to the differences in patterns of drug use between communities within the country [17]. In Muheza District Hospital, for instance, SP was implemented as first-line drug in children less than 5 years of age since 1984 [44].

The frequency of triple-*dhfr*/double-*dhps *mutants (quintuple mutation, 19.8%) depicted (Table 3) is high as compared to previous studies in Mlimba and Idete [45] which was reported as a rare event. However, the proportion of quintuple mutation in Mlimba was less than that reported by Pearce et al. [17] in Hai and Pare areas of Northern Tanzania (30 – 63%). The proportion of quintuple mutation was also less than that generally reported by Jelinek et al. [35] (42.9%) in East Africa, and that by Bwijo et al. [46] in Maonga and Chimbala villages of Salima District, Malawi (78%). The lower frequency of quintuple mutations (19.8%) observed in this study as compared to other reported mutations in Tanzania and other areas of Africa could partly be attributable to the early development of resistance in those areas. In Malawi for instance, the study was carried 7 years after introduction of SP as a first-line drug while our study was carried shortly (2 years) after introduction of SP as an interim first-line drug in Tanzania, providing a relatively shorter time for selection of SP resistance mutations. In addition the low quintuple mutations can also be linked to the proportion of genotypes at similar locus possessing mixed variants observed in this study. In previous studies, the mixed variants were not reported. The random selection of these mutations might generally, be a consequent of country-wide use of SP as a second-line antimalarial drug several years before it was implemented as an interim first-line drug by August 2001.

The prevalence of double *dhps *mutation (Table 3), which is considered to be a prerequisite for resistance to sulphonamides [7], was found to be 6× that reported by Mugittu et al. [15] in the same area. This proportion (19.8%) was also higher than that reported in Kyela and Masasi areas of Tanzania but nearly equal to that reported in Butimba but less than that reported in Mkuzi areas of Tanzania by Mugittu et al. This might be attributed to the effect of septrin, an antifolate extensively used in Tanzania as antibiotic agent, indirectly and preferentially selecting double mutations on the *dhps *locus [39]. Similar effects can result from septrin, which is essentially similar to co-trimoxazole in composition as previously stated. Both are antifolates basically composed of trimethoprim-sulphamethoxazole (TS) with similar effects to SP [46,47]. Observation also showed that 52.1% of infections harboured at least one mutation on the *dhps *locus (Table 3) reflecting that probably there is high effect or prevailing use of septrin in the area. This can also be associated with long-term abuse of the drug, but also could be indicative of the differences in the generic drugs available in the country manufactured by different companies which might have different bioavailability and therapeutic values. The high proportion of double *dhps *mutations when coupled with triple mutations in *dhfr *can result into quintuple mutation to confer highest SP resistance.

Mixed genotypes in either *dhfr *or *dhps*, or both, were depicted in at least 68 DNA samples (Table 4). Detection of mixed genotypes in *dhfr *and *dhps *is important due to its influence on the overall proportion of point mutations in baseline samples which upon drug pressure, the wild type get cleared with mutant genotypes persisting in longitudinal follow up samples. Most recrudescent infection detected in follow up cases came from pure mutant and mixed genotypes in baseline samples as parasites with wild type genotypes are sensitive and were subsequently cleared post-medication by the drug. The variable results obtained following PCR-RFLP of follow up samples (20%) have been previously observed [25]. Infection detected during follow-up may be either an infection that previously failed to express due to presence of an abundant (detected) strain that masked the presence of minor resistant strain, thus being a recrudescent parasite. But on the other hand the detected mutant alleles during follow-up may be new infection with similar genotype as that detected pre-treatment.

## Conclusions and recommendations

In conclusions, the impact of quintuple mutation on SP resistance may be weighed down by host immunity in endemic areas although may not suggest continued use of the drug for treatment of malaria. The impact of other drugs with similar mechanisms of action used as antibiotics in selecting mutations responsible for SP resistance need be studied especially for co-trimoxazole, which is currently used as a prophylaxis against opportunistic infections in HIV-infected individuals. The information obtained in the present study will be of direct and immediate relevance to current HIV and malaria control policies in Tanzania and possibly in Africa and the universe. In addition, it will add to our basic knowledge of the molecular basis of antifolate-resistant malaria. There is a need for reviewing the policy on the use of SP as a first-line drug for treating malaria in Tanzania. In addition, despite several previous studies showing SP + Amodiaquin (SP+AQ) and SP+Artesunate (SP+AS) to cause a delay in emergence of resistance and rapid gametocyte clearance, the high SP resistance potential detected in this study suggests exclusion of SP component in future planned drug combinations for treatment of malaria. Alternative combination therapies like artemisinin-based drugs (e.g. artemisinin-lumefantrine) and short-acting antimalarials such as chlorproguanil-dapsone combination (LapDap) and atovaquone-proguanil (Malarone^®^) may be rewarding albeit thorough clinical trials are still needed to evaluate the effectiveness and possible harmful side effects of these proposed drugs. In deployment of a new antimalarial drug for treating malaria, the effect of other drugs with similar mode of action to drug, used in treating other infections than malaria have to be considered to preclude the possibilities of early development of resistance to the target drug due to cross-resistance. The findings from this study imply that *in vivo *studies be further carried out to confirm that the high frequency of SP resistance alleles is indicative of treatment failure. Improvement of health services with adequate drugs and skilled medical staffs from village levels may reduce uncontrolled and inappropriate use of the drug, consequently reducing the chances of selecting SP resistance mutations against malaria.

## Authors' contributions

**EVM: **Conception and designing the study; carried out the molecular genotyping, acquisition, statistical analysis, interpretation of data and drafting the manuscript.

**BMM: **Conceived of the study, participated in its design and coordination and helped to draft the manuscript and in revising it critically for important intellectual content.

**ALM: **Conceived of the study, and participated in its design and helped to draft the manuscript, also helped in revising it critically for important intellectual content.

**STB: **Coordinated and helped to draft the manuscript and revising it critically for important intellectual content.

**TBN: **Participated in drafting the manuscript and critically revising the manuscript for intellectual content.

**HM: **Original conception and designing of the study and critical review for important intellectual content.

**NB: **All authors read and approved the final manuscript for submission.
